# Quantity of virulent fowl adenovirus serotype 1 correlates with clinical signs, macroscopical and pathohistological lesions in gizzards following experimental induction of gizzard erosion in broilers

**DOI:** 10.1186/1297-9716-44-38

**Published:** 2013-05-24

**Authors:** Beatrice Grafl, Dieter Liebhart, Ayse Günes, Patricia Wernsdorf, Franz Aigner, Josef Bachmeier, Michael Hess

**Affiliations:** 1 Department for Farm Animals and Veterinary Public Health, Clinic for Avian, Reptile and Fish Medicine, University of Veterinary Medicine, Veterinaerplatz 1, Vienna, A-1210, Austria; 2 Brüterei Süd; ZN der BWE-Brüterei Weser-Ems GmbH & Co. KG, Peter-Henlein-Straße 1, Regenstauf, D-93128, Germany

## Abstract

In the present study day-old specific-pathogen-free (SPF) and commercial broilers with maternally derived fowl adenovirus serotype 1 (FAdV-1) antibodies were orally infected with a European “pathogenic” FAdV-1, isolated from broilers showing signs of gizzard erosion. During the experiment, broilers were observed and weighed daily up to 17 days post infection (dpi). Clinically, both infected groups showed significant decrease of weight compared to respective negative control groups. Birds were examined by necropsy at 3, 7, 10, 14 and 17 dpi. Pathological changes in the gizzards were noticed in both experimentally infected groups from 7 dpi onwards. Macroscopically, erosion of the koilin layer and inflammation or ulceration of the gizzard mucosa were observed. Histologically, presence of FAdV-1 in intranuclear inclusion bodies of degenerated glandular epithelial cells was demonstrated by *in-situ* hybridization and inflammatory cell infiltration of the lamina propria, submucosa and muscle layer was detected. Tissue samples were investigated by a recently developed real-time PCR and the viral DNA load was calculated from gizzard, liver, spleen and cloacal swabs with the highest amounts of FAdV-1 DNA found in the gizzard. For the first time, successful reproduction of clinical signs in broilers as well as pathological lesions in the gizzard were achieved with a European FAdV-1 isolate displaying some genetic differences to so far reported virulent FAdV-1 from Japan. Furthermore, highest viral load in gizzards could be linked with macroscopical and histological lesions. Therefore, the conducted analyses provide important insights into the pathogenesis of adenoviral gizzard erosion.

## Introduction

Since the early 1990s, investigations towards the role of fowl adenovirus serotype 1 (FAdV-1) as primary pathogen in connection with outbreaks of gizzard erosion have been ongoing. The disease has been characterized by discoloration and erosion of the gizzard koilin layer and is confirmed by histological investigations documenting adenoviral intranuclear inclusion bodies in gizzard epithelial cells and/or isolation of FAdV-1 from affected gizzard samples. Tanimura et al. [1] reported adenoviral gizzard erosion for the first time from a natural outbreak in a flock of layer chickens. Subsequent reports generally originated from broiler flocks in Japan, but more recently also in Europe and Korea [2-9]. Experimental reproduction of adenoviral gizzard erosion by infection with FAdV-1 isolated from field outbreaks has been reported in specific-pathogen-free (SPF) layers as well as in commercial broilers. However, conflicting clinical data were described in these reports. While experimental infection usually induced no or only mild clinical signs, such as weight loss and anorexia [10-12], surprisingly Domanska et al. [6] reported 100% mortality in one-day-old birds.

In the past, virus detection was based on virus isolation, conventional PCR or histological techniques [13]. Recently, a real-time PCR assay for the detection and quantification of all FAdV species (FAdV-A to FAdV-E) has been established [14], facilitating an easier approach to study dynamic quantitative distribution of virulent FAdV-1 in experimentally infected chicken.

The aim of this study was i) to reproduce the clinical and pathological picture of adenoviral gizzard erosion with a European FAdV-1 isolate and ii) to investigate the viral load in different organ samples of experimentally infected broilers over time in order to ascertain connections between observed pathological lesions and the viral genome copy numbers.

## Materials and methods

### Virus

The FAdV-1 strain used in the experiment was isolated from a pool of gizzards collected from an outbreak of gizzard erosion in Germany [8]. The virus was identified as a European “pathogenic” FAdV-1, by comparing nucleic acid sequences of long and short fiber genes according to a method described by Marek et al. [5]. The virus was propagated on primary chicken embryo liver (CEL) cells and used to infect chickens at the fifth passage.

### Animal experiment, clinical signs and cloacal swab samples

SPF broiler eggs were obtained from Animal Health Service, Deventer, The Netherlands. Commercial broiler eggs were obtained from a FAdV-1 seropositive broiler breeder flock. Following hatching, the birds were divided into two groups, respectively. Each group comprised 25 birds. The birds were housed separately in isolator units under negative pressure (Montair Andersen bv, HM 1500, Sevenum, The Netherlands) until termination of the study. The broilers were individually marked by Swiftack™ (Heartland Animal Health, Inc., Missouri, USA). Feed and water were provided *ad libitum* during the animal experiment. One group of SPF broilers (NSPFB) and one group of commercial broilers (NCB) were kept as negative controls and none of the birds were infected at any time. In one group of SPF broilers (SPFB) and one group of commercial broilers (CB), day-old birds were inoculated orally with 0.1 mL of the virulent FAdV-1 isolate, each bird receiving 10^7.8^ mean tissue culture infective dose (TCID_50_). The birds were monitored daily for any clinical signs. The body weight of all chickens was measured on the first day of life and at 3, 7, 10, 14 and 17 days post infection (dpi). In addition, at each of these sampling points 5 birds per group were euthanized and necropsied. Cloacal swabs were taken at intervals of 3 to 4 days from the 5 birds per group that lived until 17 dpi. Blood samples were taken weekly from all birds.

The experiment was approved by the institutional ethics committee, the Advisory Committee for Animal Experiments (§12 of the Law for Animal Experiments, Tierversuchsgesetz – TVG) and the Federal Ministry for Science and Research under license no. 68.205/0179-II/10b/2009.

### Post mortem examination

During post mortem examination gizzards in particular were investigated for pathological changes. A scoring scheme from Nakamura et al. [12] was applied to assess gross lesions of the koilin layer. Changes of the gizzard mucosa were investigated and scored in the same manner: no lesions = 0; mild lesions (less than one-third of the koilin layer/gizzard mucosa was affected) = 1; moderate lesions (one-third to one-half of the koilin layer/gizzard mucosa was affected) = 2 and severe lesions (more than one-half of the koilin layer/gizzard mucosa was affected) = 3.

For virological and histological investigations, tissue samples of the gizzard, liver and spleen were collected.

### Histology

Samples of gizzard, liver and spleen were fixed in 3.5% neutral buffered formalin and then embedded in paraffin for histological investigations. Using a Microm HM 360 microtome (Microm Laborgeräte GmbH, Walldorf, Germany) tissue slices of 3 μm were prepared and routine staining using hematoxylin and eosin (H&E) was performed. The scoring scheme from Nakamura et al. [12] was used to assess intranuclear inclusions in the gizzard glandular epithelium: no lesions = 0; mild lesions (1 to 10 inclusions in one section) = 1; moderate lesions (10–20 inclusions in one section) = 2 and severe lesions (more than 20 inclusions in one section) = 3.

In order to demonstrate FAdV-1 DNA in paraffin-embedded tissue samples *in-situ* hybridization was performed according to a protocol described earlier [8], using a DNA probe based on the FAdV-1 long fiber gene.

### Virus isolation and SYBR Green based real-time PCR

Cloacal swabs were placed in 1 mL of an antibiotics-phosphate buffered saline (PBS) solution (1 mg/mL streptomycin and 100 000 IU/mL penicillin). Tissue samples from gizzard, liver and spleen of the birds were homogenized in antibiotics-PBS solution. Samples were filter sterilized using syringe filters with a pore size of 0.2 μm (VWR, Vienna, Austria). Cloacal swab samples and tissue homogenates (20%) were used for virus isolation and for DNA extraction followed by real-time PCR.

For virus isolation CEL cell cultures were prepared according to a method described by Schat and Sellers [15] with some modifications. 100 μL of a cloacal swab sample or tissue homogenate were inoculated on nearly confluent CEL cells. Each sample was passaged up to three times or until a cytopathic effect was observed. A sample was considered negative when no cytopathic effect was noticed after three blind passages.

Viral DNA was extracted from 100 μL of the homogenized organ samples and from cloacal swabs with DNeasy Blood & Tissue Kit (Qiagen, Hilden, Germany) according to manufacturer’s instructions. The SYBR Green based real-time PCR was performed as described recently in order to quantify virus DNA in sample material [14]. Briefly, primers were designed to anneal within the highly conserved 52 K region. Using a double-stranded DNA-binding dye method with a Rotor-Gene SYBR Green PCR kit (Qiagen, Hilden, Germany) the real-time PCR was performed on a Rotor-Gene Q thermal cycler (Qiagen, Hilden, Germany). No template controls were included throughout sample preparation and PCR runs, in order to avoid cross contamination. To confirm real-time PCR product specificity melting curve analysis was done and amplification products were separated by electrophoresis in a 2% agarose gel. Threshold cycle (C_T_) values of investigated samples were compared with a well-defined standard curve and the number of copies of FAdV DNA per reaction mixture was calculated.

### Serology

Prior to testing, all sera were inactivated for 30 min at 56°C in a thermomixer (Eppendorf, Vienna, Austria). The serum samples were tested for FAdV-1 specific antibodies by virus-neutralization test (VNT). The VNT was performed according to a constant virus diluted serum method using 100 TCID_50_/100 μL of the FAdV-1 strain used in the animal experiment. Respective titers were calculated according to Kärber [16]. An antibody titer below or equal to 3 log_2_ was regarded as negative.

### Statistical analysis

The average body weight of orally infected broilers and uninfected controls at 0, 3, 7, 10, 14 and 17 dpi was recorded and analyzed by the student *t*-test. Statistical differences with *P* < 0.05 were considered to be significant. Data were analyzed with the statistical software package SPSS Version 17 (IBM SPSS Statistics; IBM Corporation, Somer, New York, USA).

## Results

### Clinical signs and weight gain

**Figure 1:**
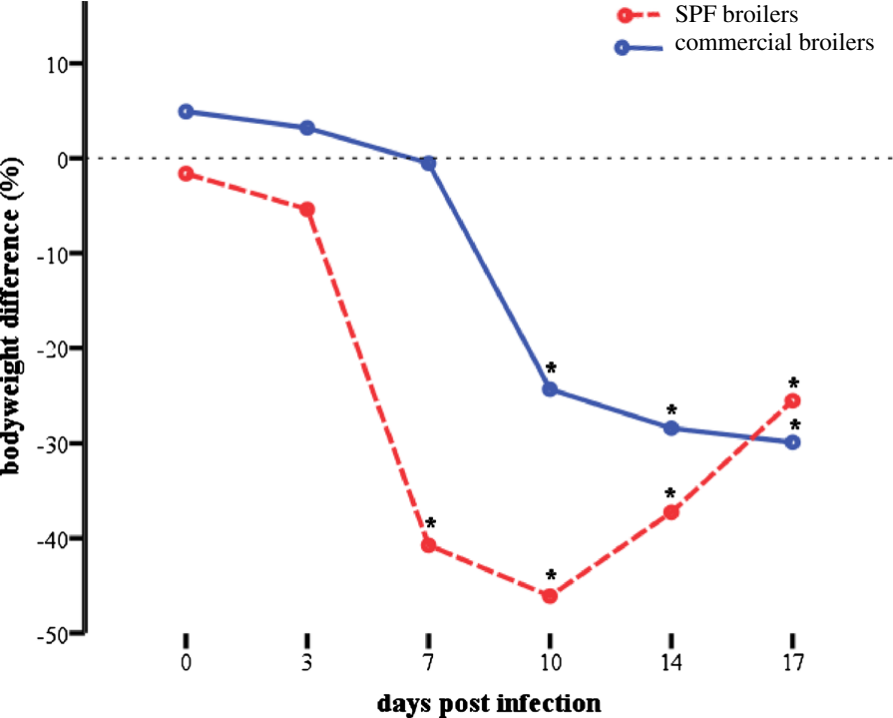
**Graphical illustration of decreased weight gain.** Difference of body weight (%) from experimentally infected specific-pathogen-free (SPFB) and commercial (CB) broilers to respective negative control groups at 0, 3, 7, 10, 14 and 17 days post infection. Asterisks indicate a significant difference from negative control group (*P* < 0.05).

Both orally infected groups, SPFB and CB, showed decreased weight gain in comparison to the respective uninfected control groups (Figure 1). SPFB showed significantly decreased weight from 7 dpi until termination of the experiment (*P* < 0.04). CB showed significantly decreased weight gain from 10 dpi onwards (*P* < 0.01). No further clinical signs were observed in any of the broilers.

### Gross pathology

None of the chickens in groups SPFB or CB showed macroscopic lesions of the koilin layer or the gizzard mucosa at 3 dpi. Gizzard erosions, characterized by discoloration and ablation of the koilin layer, were noticed from 7 dpi onwards, as given in Table 1. In SPFB, the gross lesions of the koilin layer were most severe at 10 dpi, reaching an average lesion score of 2.4. In CB, the most severe lesions of the koilin layer were documented at 14 dpi with a mean lesion score of 2.2. Similarly, inflammation and ulceration of the gizzard mucosa beneath the degenerative koilin layer appeared at 7 dpi and persisted until termination of the experiment at 17 dpi (Table 1). The most distinct ulcerations were found in group SPFB at 7 dpi and in group CB at 10 dpi, reaching a mean lesion score of 1.8 and 1.6, respectively. Gizzards of birds from negative control groups did not present pathological changes. No pathological lesions were found in any other organ.

**Table 1:** Pathological changes in the gizzard

		**Macroscopical lesions**		**Histological lesions**	
**Group**	**dpi**	**Koilin layer**	**Gizzard mucosa**	**Inclusion bodies in glandular epithelium**	***In-situ *hybridization**
SPFB	3	0/5 (0.0)^a^	0/5 (0.0)	0/5 (0.0)	0/5
	7	5/5 (1.8)	5/5 (1.8)	5/5 (2.6)	5/5
	10	5/5 (2.4)	5/5 (1.0)	1/5 (0.2)	1/5
	14	5/5 (1.8)	2/5 (0.4)	0/5 (0.0)	NE^b^
	17	4/5 (0.8)	3/5 (0.6)	0/5 (0.0)	NE
CB	3	0/5 (0.0)	0/5 (0.0)	0/5 (0.0)	0/5
	7	3/5 (0.6)	3/5 (0.8)	4/5 (1.6)	4/5
	10	4/5 (1.2)	5/5 (1.6)	5/5 (2.0)	5/5
	14	5/5 (2.2)	4/5 (0.8)	0/5 (0.0)	NE
	17	5/5 (1.6)	4/5 (0.8)	0/5 (0.0)	NE

Specific-pathogen-free (SPFB) and commercial (CB) broilers orally infected with virulent FAdV-1 at 3, 7, 10, 14 and 17 days post infection (dpi).

^a^ No. birds positive/no. birds examined (mean lesion scores in parenthesis; no lesion = 0, mild lesions = 1, moderate lesions = 2 and severe lesions = 3).

^b^ NE – not examined.

### Histology

**Figure 2:**
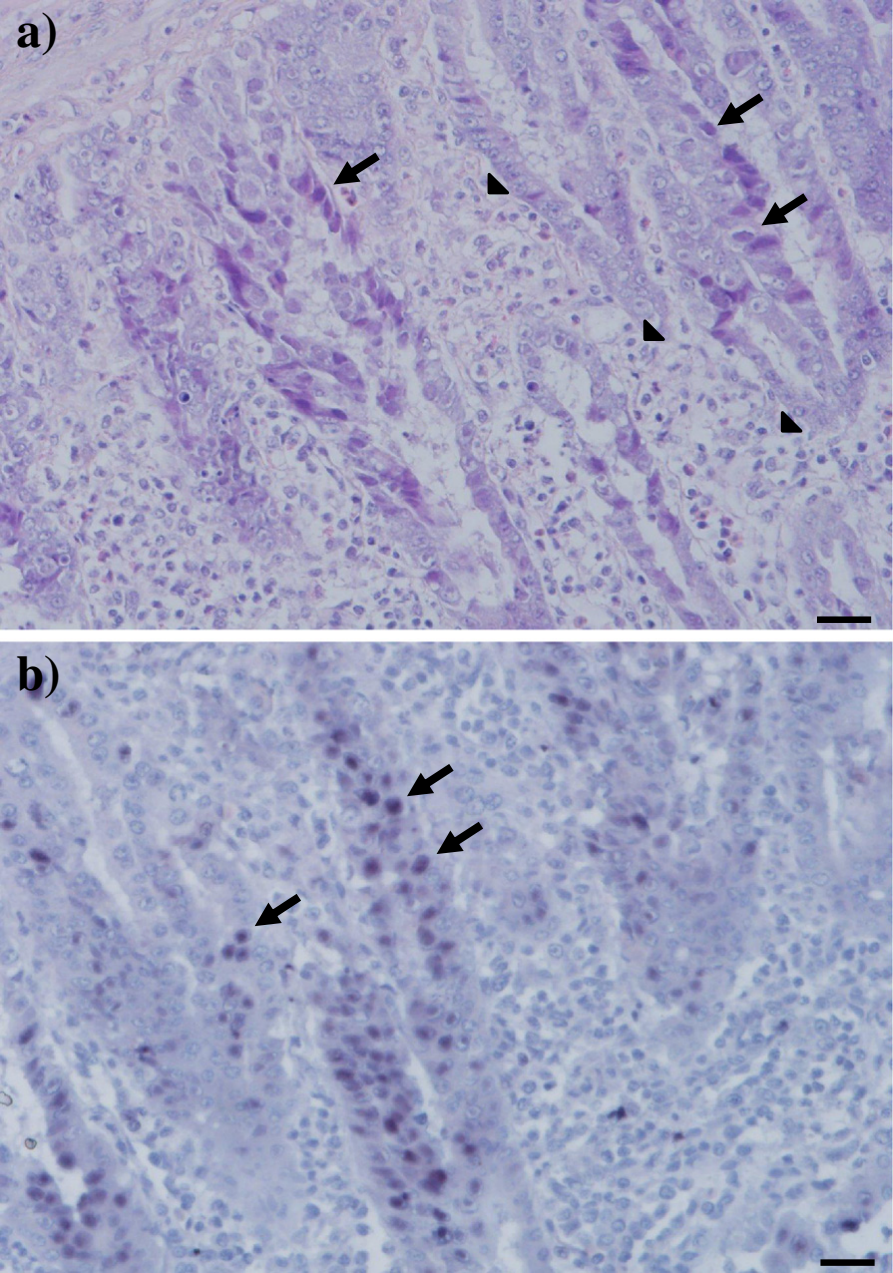
**Histopathological lesions in the gizzard.** Gizzard from a commercial broiler experimentally infected on the first day-of-life with virulent FAdV-1. **a**) Expansion of the lamina propria mucosa by mixed population of inflammatory cells (arrowheads). Basophilic intranuclear inclusion bodies in degenerated epithelial cells (arrows). Haematoxilin & eosin staining. Bar = 20 μm. **b**) *In-situ* hybridization of the same gizzard sample with positive signal for FAdV-1 DNA in epithelial cells (arrows). Bar = 20 μm.

From 7 dpi onwards, gizzards of orally infected broilers from groups SPFB and CB showed histological changes, such as necrosis, degeneration and disappearance of epithelial cells accompanied with mild to severe inflammation of the lamina propria mucosa, the submucosa and the muscle layer. Within and near such areas, multiple basophilic intranuclear inclusion bodies were observed in glandular epithelial cells at 7 and 10 dpi (Figure 2a). In group SPFB they were found more frequently at 7 dpi and in group CB at 10 dpi (Table 1). FAdV-1 DNA was demonstrated in affected gizzards, particularly in intranuclear inclusion bodies, by *in-situ* hybridization (Figure 2b). No lesions or intranuclear inclusion bodies were found in the other investigated organs or in organ samples taken from the negative control groups.

### Virus isolation and SYBR Green based real-time PCR

**Figure 3:**
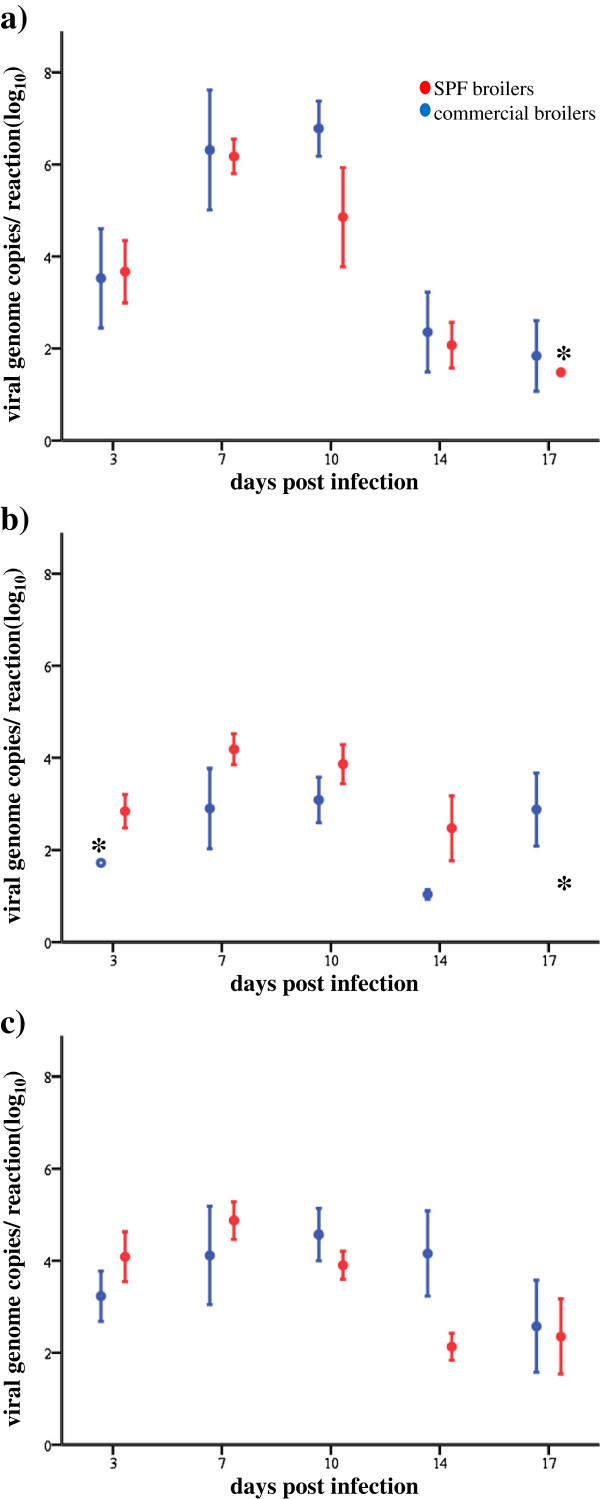
**Graphical illustration of the viral load.** Viral genome copies per reaction (log_10_) from **a**) gizzard, **b**) liver and **c**) cloacal swabs calculated by real-time PCR from specific-pathogen-free (SPF) and commercial broilers orally infected with virulent FAdV-1 at 3, 7, 10, 14 and 17 days post infection. Asterisks indicate some samples that turned out positive with specific melting curve analysis but viral genome copies per reaction could not be calculated successfully.

Cloacal swabs and organ samples collected from broilers experimentally infected with FAdV-1 were investigated by cell culture and real-time PCR. In SPFB, FAdV-1 was recovered from cloacal swabs, gizzard, liver and spleen by cell culture and real-time PCR (Table 2). Virus excretion and presence of live virus in internal organs were noticed until 10 dpi. Viral genome copy numbers were determined and mean values for each time point post infection were calculated. The highest number of excreted FAdV-1 as well as the maximum viral load in gizzard and liver samples was found at 7 dpi (Figure 3). In CB, live virus was recovered from cloacal swabs, gizzard and liver by cell culture, whereas spleen samples were all negative. However, viral genome copy numbers could be determined from cloacal swabs, gizzard, liver and spleen (Table 2) and the highest viral loads were calculated at 10 dpi (Figure 3).

**Table 2:** Virus isolation and nucleic acid detection in samples obtained from organs and cloacal swabs

**Group**	**dpi**	**Cloacal swabs**		**Gizzard**		**Liver**		**Spleen**	
		**VI**	**Real-time PCR**	**VI**	**Real-time PCR**	**VI**	**Real-time PCR**	**VI**	**Real-time PCR**
SPFB	3	5/5 ^a^	5/5	5/5	5/5	4/5	5/5	0/5	NE ^b^
	7	5/5	5/5	5/5	5/5	5/5	5/5	4/5	5/5
	10	5/5	5/5	5/5	5/5	5/5	5/5	1/5	2/5
	14	0/5	5/5	0/5	4/5	0/5	5/5	0/5	NE
	17	0/5	5/5	0/5	3/5	0/5	3/5	0/5	3/5
CB	3	5/5	5/5	3/5	5/5	0/5	3/5	0/5	NE
	7	4/5	5/5	5/5	5/5	1/5	5/5	0/5	4/5
	10	5/5	5/5	5/5	5/5	2/5	5/5	0/5	4/5
	14	4/5	5/5	1/5	5/5	0/5	4/5	0/5	NE
	17	0/5	5/5	1/5	5/5	0/5	5/5	0/5	1/5

Samples from specific-pathogen-free (SPFB) and commercial (CB) broilers orally infected with a virulent FAdV-1 were investigated at 3, 7, 10, 14 and 17 days post infection (dpi) by virus isolation (VI) and/or real-time PCR.

^a^ No. of positive samples/no. of tested samples.

^b^ NE – not examined.

### Serology

**Figure 4:**
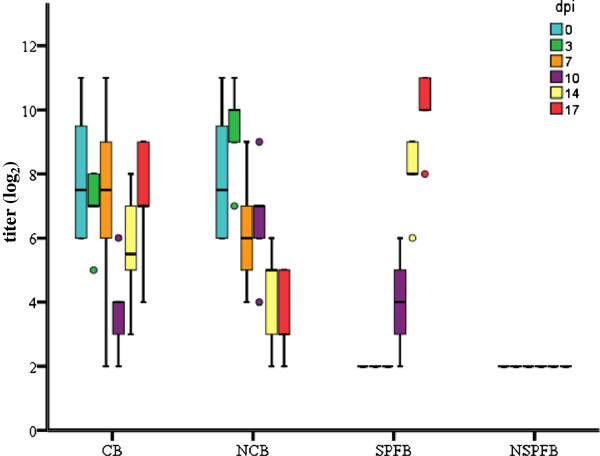
**Box plot presentation of virus neutralization test.** Titers (log_2_) of FAdV-1 specific antibodies from experimentally infected specific-pathogen-free (SPFB) and commercial (CB) broilers as well as respective negative control groups (NSPFB & NCB) at 0, 3, 7, 10, 14 and 17 days post infection (dpi) are shown. Titers ≤ 3 log_2_ are considered negative.

In group SPFB, virus neutralizing antibodies were present in orally infected chickens from 10 dpi onwards. Birds from group NSPFB were serologically negative throughout the experimental investigations (Figure 4).

On the first day of life commercial broilers showed maternal antibodies against FAdV-1 with a titer of 7.93 ± 1.59 log_2_. Neutralizing antibodies declined to a titer of 3.80 ± 1.48 log_2_ at 10 dpi and then proceeded to increase to a titer of 7.20 ± 2.04 log_2_ at 17 dpi. Titers in group NCB steadily declined until 17 dpi to a titer of 3.60 ± 1.34 log_2_ (Figure 4).

## Discussion

The FAdV-1 strain used in this experimental study was isolated from an outbreak of gizzard erosion in Germany [8]. This virus was grouped together with European “pathogenic” FAdV-1, according to nucleic acid sequences of the long and short fiber. These European “pathogenic” strains differ in their PCR - restriction fragment length polymorphism (RFLP) patterns from previously reported and investigated Japanese “pathogenic” FAdV-1 as described by Marek et al. [5]. Experimental oral infection of day-old SPF broilers as well as commercial broilers with maternal FAdV-1 antibodies with this European FAdV-1 isolate resulted in clinical signs, macroscopical and histological gizzard lesions comparable to those described from the natural outbreak, demonstrating the pathogenicity of this strain.

In the present study, experimentally infected broilers with adenoviral gizzard erosion showed clinical signs of growth retardation, reflecting production data collected from the natural outbreak. Similar to this, decreased weight gain was described by Okuda et al. [11] in an experimental study of 1-week-old and 3-week-old commercial broilers. The generated data as well as information collected from field outbreaks [6,8,9] confirm that growth retardation in the course of FAdV-1 induced gizzard erosion may present a serious problem for the economic success of broiler production. Similar to the majority of other experimental studies set up to reproduce gizzard erosion, no other clinical signs were observed in the present investigation. This is in direct contrast to the experimental work reported by Domanska-Blicharz et al. [6], who noticed 100% mortality in day-old birds infected with a Polish FAdV-1 strain by nasal and ocular routes.

Although experimental reproductions of FAdV-1 induced gizzard erosion have been described [10,11], this is the first study aiming to link viral load in gizzards beside other tissue samples and cloacal swabs with the appearance and severity of gizzard lesions. In order to achieve this, a recently developed real-time PCR was applied [14], offering certain advantages in comparison to reported cell-culture based titrations methods and conventional PCR to detect FAdV-1. Quantifying the viral load in sample material by tissue culture requires special facilities and is less suitable for processing a large number of samples. Furthermore, these investigations may present an incomplete picture, considering the high sensitivity of the real-time PCR, which was demonstrated in a previous study investigating viral shedding of all FAdV species in experimentally infected birds [14]. In the present study viral DNA was detected in nearly all samples investigated throughout the experiment, whereas virus isolation on cell culture detected fewer numbers of positive samples. This result underlines the applicability of real-time PCR for investigating organ samples. However, variations in sample preparation limits the comparison between data obtained for organ samples and those recorded for cloacal swabs.

In the present study the highest viral genome copies per reaction were found in the gizzard. Furthermore, in accordance to previous experimental studies of FAdV-1 induced gizzard erosion [17], successful virus re-isolations from organ samples were shown predominantly from the gizzards. Comparing results obtained by real-time PCR with those from virus isolation, it can be concluded that virus isolation may succeed more easily from samples with higher amounts of viral DNA as they represent higher amounts of viable virus. Overall, the calculated viral load from the gizzards was followed by similar quantitative and chronological distribution patterns in other investigated samples. Nevertheless, even the highest genome copy numbers in other investigated organs did not reach the numbers calculated for gizzard samples. Pathological changes and detection of the virus in the gastrointestinal system, particularly in the gizzard, in the course of a FAdV-1 infection are contrary to other documented FAdV infections, such as inclusion body hepatitis and hydropericardium/hepatitis syndrome, both targeting the liver with corresponding tissue destruction [18]. A putative FAdV-1 cell receptor in gizzard mucosal cells, described by Taharaguchi et al. [19], may explain this tissue tropism of virulent FAdV-1.

Overall, the levels of viral DNA in liver and gizzard achieved their highest numbers in groups SPFB at 7 dpi and CB at 10 dpi. At the same time, onset of clinical signs was observed and macroscopical lesions in the gizzard mucosa appeared most severe. Additionally, histolopathological changes, in particular numbers of intranuclear inclusion bodies in the gizzard epithelial cells, were assessed concurrently with the highest scores. However, erosion of the gizzard koilin layer was found to be most distinct at later dates, when viral load was already declining and intranuclear inclusion bodies were found far less frequent or absent. Based on these data, the development of adenoviral gizzard erosion reflects a cascade of reactions in which infection and subsequent necrosis of mucosal epithelial cells play a crucial role [12], even though the precise mechanism needs to be resolved.

In accordance with a previous experimental study [11], our results underline that the development of gizzard erosion is largely due to a localized FAdV-1 infection in the gizzard, with the inability of maternal antibodies to prevent the disease. Nonetheless, the presence of maternal antibodies in experimentally infected commercial broilers delayed the formation of gizzard erosion for a short time compared to the progress of infection in SPF broilers, suggesting that viremia may play a role in gizzard erosion pathogenesis as well. In contrast to previous experimental studies [7,10,11], the systemic distribution of live virus to the liver was confirmed in SPF and commercial broilers. In the spleen viable FAdV-1 could only be detected in SPF broilers which might reflect an influence of maternal antibodies. The systemic distribution of the virus to the liver and spleen was confirmed in the majority of infected birds by real-time PCR, with a tendency of lower numbers of viral DNA in samples of commercial broilers. Nevertheless, similar to previous experimental studies [11,12,20], neither macroscopical nor histopathological changes were documented in the liver or spleen, no adenoviral inclusion bodies were observed and no viral DNA was demonstrated by *in-situ* hybridization, suggesting that replication may not be very efficient in these organs, if it takes place at all.

The actual study also demonstrates that diagnosis of adenoviral gizzard erosion based on macroscopical lesions of the koilin layer, virus isolation or the appearance of typical adenoviral inclusion bodies alone may be inconclusive. Ono et al. [21] postulated that cellular infiltration in the lamina propria as well as mononuclear infiltration in the perivascular tissue of submucosa and muscle layer, both of them were also observed in the course of our experimental study, may aid in histological diagnostics of adenoviral gizzard erosion. Furthermore, the actual study demonstrates that the highly sensitive real-time PCR is a useful tool to complete the range of diagnostics in the course of adenoviral gizzard erosion.

To summarize, for the first time experimental infection with a European FAdV-1 succeeded in reproducing typical clinical signs and pathological changes of adenoviral gizzard erosion. Both, SPF broilers and commercial broilers with homologous maternal antibodies, developed typical gizzard lesions as well as clinical signs of decreased weight gain, confirming that maternal antibodies have no protective effect in the course of an infection with a virulent FAdV-1. Investigations of samples from experimentally infected broilers over time with a recently developed real-time PCR showed that maximum viral load in the gizzard and other investigated samples coincided with the onset of clinical signs as well as maximum macroscopical and histological lesions, altogether helpful to elucidate the pathogenesis of the disease.

## Abbreviations

SPF: Specific-pathogen-free; FAdV-1: Fowl adenovirus serotype 1; Dpi: Days post infection; CEL: Chicken embryo liver; NSPFB: Negative control group of SPF broilers; NCB: Negative control group of commercial broilers; SPFB: Group of experimentally infected SPF broilers; CB: Group of experimentally infected commercial broilers; H&E: Hematoxilin and eosin; PBS: Phosphate buffered saline; VNT: Virus neutralization test; TCID50: Mean tissue culture infective dose; CT: Threshold cycle; RFLP: Restriction fragment length polymorphism.

## Competing interests

The authors declare that they have no competing interests.

## Authors’ contributions

MH, FA, JB and BG participated in the design of the study. BG carried out the animal experiment and cell culture techniques and drafted the manuscript. DL and PW carried out histological investigations. AG carried out real-time PCR investigations. All authors read and approved the final manuscript.
